# Category-Specific Processing of Scale-Invariant Sounds in Infancy

**DOI:** 10.1371/journal.pone.0096278

**Published:** 2014-05-08

**Authors:** Judit Gervain, Janet F. Werker, Maria N. Geffen

**Affiliations:** 1 Laboratoire Psychologie de la Perception, CNRS, Paris, France; 2 Université Paris Descartes, Sorbonne Paris Cité, Paris, France; 3 Department of Psychology, University of British Columbia, Vancouver, British Columbia, Canada; 4 Department of Otorhinolaryngology: HNS, University of Pennsylvania, Philadelphia, Pennsylvania, United States of America; University of Salamanca- Institute for Neuroscience of Castille and Leon and Medical School, Spain

## Abstract

Increasing evidence suggests that the natural world has a special status for our sensory and cognitive functioning. The mammalian sensory system is hypothesized to have evolved to encode natural signals in an efficient manner. Exposure to natural stimuli, but not to artificial ones, improves learning and cognitive function. Scale-invariance, the property of exhibiting the same statistical structure at different spatial or temporal scales, is common to naturally occurring sounds. We recently developed a 3-parameter model to capture the essential characteristics of water sounds, and from this generated both scale-invariant and variable-scale sounds. In a previous study, we found that adults perceived a wide range of the artificial scale-invariant, but not the variable-scale, sounds as instances of natural sounds. Here, we explored the ontogenetic origins of these effects by investigating how young infants perceive and categorize scale-invariant acoustic stimuli. Even though they have several months of experience with natural water sounds, infants aged 5 months did not show a preference, in the first experiment, for the instances of the scale-invariant sounds rated as typical water-like sounds by adults over non-prototypical, but still scale-invariant instances. Scale-invariance might thus be a more relevant factor for the perception of natural signals than simple familiarity. In a second experiment, we thus directly compared infants' perception of scale-invariant and variable-scale sounds. When habituated to scale-invariant sounds, infants looked significantly longer to a change in sound category from scale-invariant to variable-scale sounds, whereas infants habituated to variable-scale sounds showed no such difference. These results suggest that infants were able to form a perceptual category of the scale-invariant, but not variable-scale sounds. These findings advance the efficient coding hypothesis, and suggest that the advantage for perceiving and learning about the natural world is evident from the first months of life.

## Introduction

A fundamental issue in auditory development is understanding the extent to which perception of natural signals is based on inherent neural organization. According to the efficient coding hypothesis, the mammalian sensory system evolved to encode sensory information efficiently at the neuronal level. A key prediction of this hypothesis is that the neural code is optimized for natural stimuli [1], [2]. Natural signals, from visual to auditory, obey scale-invariant statistics [3], [4], [5], exhibiting the same structure when observed under different temporal or spatial scales. Neurons have been found to preferentially encode stimuli that mimic the statistical structure of natural signals, efficiently encoding scale-invariant stimuli [6], [7], [8], [9].

Scale-invariance refers to the quality whereby a feature of an object remains constant as the scale at which the object is observed changes [8], [10], [11]. A formal way to characterize scale-invariance is to measure the relation between power and frequency in the Fourier transform of a signal: if a process or a feature is scale-invariant, then its power spectrum should not change when the object is stretched or compressed [11]. This can occur if the power spectrum exhibits 1/f scaling: power scales as an inverse of the frequency. Indeed, such a distribution exists for the pixel intensities in natural scenes [4]. In audition, 1/f scaling holds for the power spectrum and the time course of the amplitude envelope of environmental sounds [3], [5].

**Figure 1:**
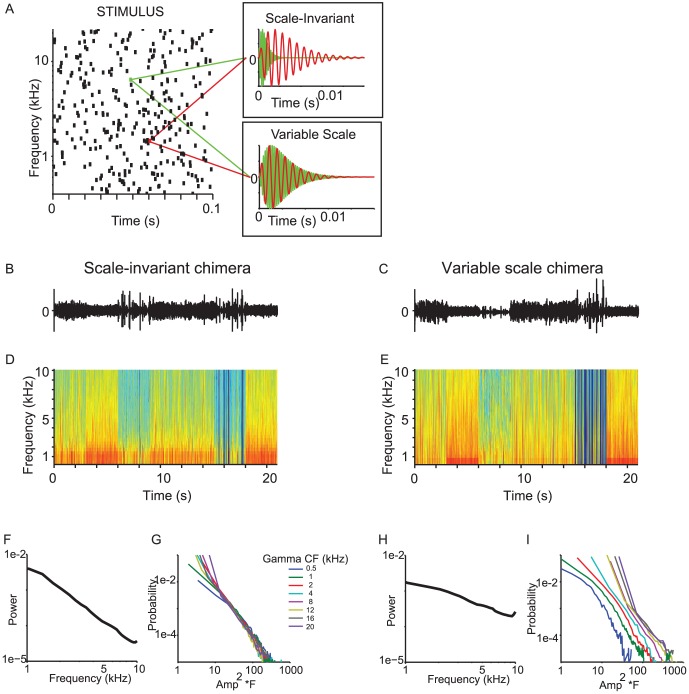
Stimulus design. A. The generative model. Left: Each bar denotes a chirp at its onset time (x-axis), center frequency (y-axis), and amplitude (height of bar). Top right: 2 chirps from scale-invariant stimulus. Bottom right: 2 chirps from variable-scale stimulus. B, C. Waveform of the 21 s chimera stimulus (used in Experiment 2). D, E. Spectrogram of the stimulus. F, H. Power as a function of frequency in the stimulus. G, I. Probability distribution of the amplitude of the gammatone transform, normalized by the center frequency, for gammatone bands at a range of frequencies (0.5–20 kHz). B, D, F: scale-invariant stimuli. C, E, H, I: variable-scale stimuli.

Recently, we identified an even narrower definition of scale-invariance for auditory signals, and demonstrated its perceptual relevance [12]: when the waveform of the recording of running water was stretched or compressed, we found that its statistical structure remained the same. Therefore, scale-invariance of water sounds was manifested at several levels: not just in the 1/f relation for amplitude modulation within spectral channels, but across spectro-temporal channels [12]. To directly examine the effect of such scale-invariant structure on auditory perception, we created a generative model of water sounds (Figure 1A, B, D) as a superposition of randomly spaced chirps spanning a wide range of frequencies [12]. Each chirp was a sinusoid enveloped in a gamma tone [13], defined by its frequency, amplitude and cycle constant of decay, *Q*. Adult listeners perceived the sounds generated by this model as natural when the temporal structure of each chirp scaled relative to its center frequency for a specific range of *Q*, but not if chirps within different spectral bands varied in the scale of temporal structure relative to their center frequency [12]. Within the category of sounds identified as natural, adults' subjective description ranged from light rain through a dripping tap to ocean waves, for sounds generated using different values for *Q* and the rate of the chirps, suggesting that the generative model is able to reproduce natural water instances in different forms [12]. When scale-invariance across spectral bands was violated (while preserving all other parameters for chirps: Figure 1A, C, E) and the temporal structure of the chirps was constant irrespective of the frequency [12], adults did not perceive the sounds as natural. We note that in these two sets of stimuli, the 1/f relation holds for the power of the signal within each of the spectral bands (Figure 1F, H). However, the slope of the 1/f relation differs, with the scale-invariant stimuli exhibiting steeper slope, and variable-scale stimuli exhibiting more gradual slope. The difference in scaling relation across spectral bands is apparent in examining the histogram of the amplitude distribution of the gamma transform in different spectral bands. For scale-invariant sounds, this distribution is the same, when normalized by the center frequency of the gamma transform (Figure 1G). By contrast, for variable-scale sounds, this distribution varies across the different channels (Figure 1I).

The ontogenetic origins of this effect and of efficient coding in general remain largely unknown. Thus in this study, we explored how infants categorize and learn about the natural world by testing their ability to categorize scale-invariant versus variable-scale sounds. If our sensory system has evolved, rather than been sculpted by experience, to efficiently encode the statistics of the natural world, an advantage for perceiving and learning about natural over unnatural stimuli should be present in early infancy.

Water sounds are among the first natural sounds infants encounter. Hence, it is likely that many water sounds, including those characterized by adults as ‘dripping water’, ‘rain’ or ‘light shower’, will be familiar to infants. To ensure that infants could not classify our stimuli simply on the basis of familiarity, but instead would do so on the basis of scale-invariance, we first tested whether infants prefer the scale-invariant sounds that adults had rated as most water-like over other scale-invariant sounds that had been rated as less typical [12]. These highly prototypical instances of scale-invariant water sounds (for which *Q*< = 3.1) are likely more familiar to infants than the untypical ones. It is thus possible that infants will prefer the more typical water-like over the atypical scale-invariant sounds, just as they do familiar (i.e. native) vs. unfamiliar speech sounds [14], [15], [16], [17], and that this preference for familiarity could drive discrimination performance.

## Experiments

### Experiment 1: Familiar vs novel scale-invariant sounds

#### Participants

Fourteen healthy infants (mean age: 5 months 4 days, range: 4 months 19 days–5 months 14 day; 7 females) from Vancouver participated. Five additional infants did not complete the study due to fussiness or crying. This research was approved by the Human Ethics Review Board of the University of British Columbia. Informed consent was obtained from the infants' parents in writing prior to participation. A copy of the consent form was given to the parent and the original was saved by the research team.

#### Materials and Methods

We used two sets of synthetic scale-invariant sounds taken from the adult psychophysics experiment of Geffen et al. [12]. Both sets were scale-invariant sounds generated by our model [12], but for one set (‘familiar’), we selected sounds that adults judged the most natural (ratings around 5 on a 1–7 scale, where an actual recording of a tropical brook was rated 5.3) and qualitatively described as prototypical occurrences of water (e.g. “rain”, “river”, “tap dripping” etc.), whereas for the other set (‘novel’), we chose sounds that adults judged the least natural (ratings below 2) and rarely described as being naturally occurring water. Four sounds, lasting 28 s, were thus chosen from the adult material for each category, with respective mean rates of 53 Hz/Oct, 530 Hz/Oct, 5300 Hz/Oct and 15300 Hz/Oct. For the familiar set, the decay constant *Q* was 3.1, for the novel sounds, 8.

We used the head-turn preference procedure [18], [19] with no familiarization to directly assess preference. Infants were tested individually while sitting on a parent's lap in a dimly lit, sound-attenuated cubicle, equipped with a central light on a panel in front of the infant, and two side-lights on panels to the left and right of the infant. Parents listened to music and wore dark sunglasses to avoid influencing the infant. During the experiment, an experimenter, blind to the stimuli and seated outside the testing cubicle, monitored infants' looking behavior and controlled the stimuli. Infants were videotaped during the experiment for off-line coding.

Infants were tested in 8 test trials. Half of the trials involved ‘familiar’ synthetic scale-invariant sounds; the other half involved ‘novel’ synthetic scale-invariant sounds. Each trial started with the blinking of the central light to attract infants' attention. Once infants attended, one of the side-lights started blinking and the central light was extinguished. When infants stably fixated on the blinking side-light, the associated sound file started playing from a loudspeaker on the corresponding side. The sound file continued until the end (28 sec) or until infants looked away for more than 2 sec. After this, a new trial began. The order and side of presentation of the test trials was randomized and counter-balanced across participants in such a way that at most two consecutive trials could be of the same type.

#### Results and discussion

Infants' average looking time to familiar scale-invariant sounds was 5.71 sec (SD: 2.64); to novel scale-invariant sounds, the average looking time was 6.41 sec (SD: 3.99). A paired-sample t-test comparing looking times to the two stimulus types yielded no significant difference (t(13) = 0.6347, p = 0.537, ns.). Eight infants had longer looking times to the familiar, six to the unfamiliar sounds (two-tailed binomial test: p = 0.79). These results suggest that infants have no preference for potentially familiar over novel instances of scale-invariant sounds. In Experiment 2 we therefore directly compared the perception of scale-invariant and variable-scale water sounds generated by our model, to test whether infants can form a category of scale-invariant sounds [12].

### Experiment 2: Scale-invariant versus variable-scale sounds

In Experiment 2, we tested whether young infants can discriminate scale-invariant (‘natural’) sounds from variable-scale (‘unnatural’) ones, as observed in adults [12]. To test discrimination, we used the habituation/dishabituation procedure wherein infants are habituated to instances of one category of sounds, and tested on their recovery to a change in category [20], [21]. Recovery in looking during the test phase provides a sensitive measure of discrimination. Establishing discrimination for a specific set of sounds would support the hypothesis that infants are able to form a category from that set of sounds. Additionally, looking time during the habituation phase, when compared between the groups habituated to one category of sound vs. the groups habituated to the other category, provides an index of attention and preference, allowing comparison, albeit from a different procedure, to Experiment 1.

#### Participants

Thirty-two healthy infants (mean age: 5 months 3 days, range: 4 months 4 days–6 months 1 day; 14 females) from Paris and thirty-two healthy infants (mean age: 4 months 28 days, range: 4 months 0 days–5 months 14 days; 16 females) from Vancouver participated. 51 additional infants did not complete the study due to fussiness. This research was approved by the Human Ethics Review Board of the University of British Columbia and University Paris Descartes. Informed consent was obtained from the infants' parents in writing prior to participation. A copy of the consent form was given to the parent and the original was saved by the research team.

#### Materials and Methods

Stimuli in both the scale-invariant and the variable-scale categories were generated using the same frequency range, loudness, chirp amplitude and timing parameters. Specifically, for scale-invariant stimuli, the sound waveform *y*(*t*) was modeled as a sum of scale-invariant chirps [12] (Figure 1, top). Each chirp was modeled as a gammatone function, with parameters amplitude, frequency *f_i_*, onset time, and cycle constant of decay, *Q* drawn at random from distinct probability distributions. *f_i_* was uniform random in log-frequency space, between 400 and 20000 Hz. The number of chirps per second was determined by the mean rate *r*. The timing of the onset of each chirp was uniform random across the length of the stimulus. The amplitude of each chirp was drawn from an inverse-uniform distribution.

Two sets of forty different 21 sec stimuli were generated, each comprising a different set of values of *Q*, and *r*, chosen at random every 3 sec (Figure 1A top inset, B, D). Set 1: *Q* varied between 1 and 3.1, Set 2: Q varied between 2 and 4; for both sets, *r* varied between 53, 530 and 5340 chirps/Octave/second. Each 21 sec stimulus thus comprised 7 continuously concatenated, 3-second-long chunks, each with its own *Q* and *r* value. The resulting waveforms were normalized for the amplitude root mean square as a proxy for loudness.

For variable-scale stimuli, 2 sets of forty different 21 sec stimuli were generated as above, but for each chirp *i*, the cycle constant of decay *scaled proportionally* to the frequency: Set 1: *Q_i_* = *0.1 f_i_*, *Q_i_* = *0.001 f_i_*; Set 2: *Q_i_* = *0.01 f_i_, Q_i_* = *0.005 f_i_*; and for both sets *r* varied between 53, 530 and 5340 chirps/Octave/second, therefore matching the scale-invariable sounds in within-category variability (Figure 1A bottom inset, Figure 1C, E) but violating the scaling relation between the temporal structure of each chirp and the center frequency.

Sets 1 of each variable-scale and one scale-invariant, were used for habituation. Sets 2 of each variable-scale and scale-invariant, were used for test.

**Figure 2:**
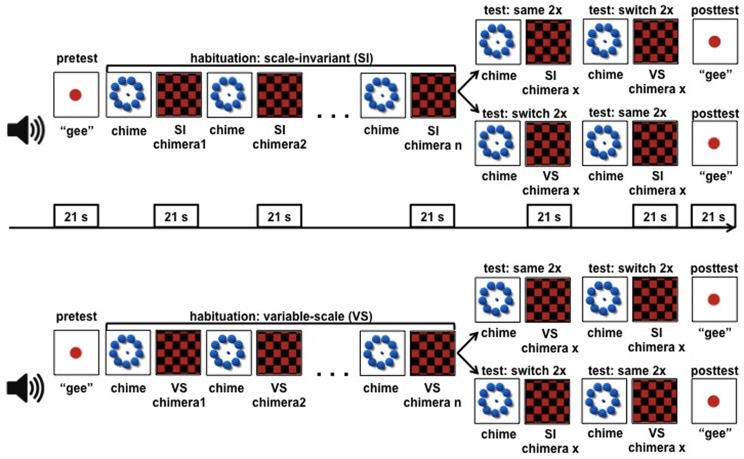
The design of Experiment 1. Half of the infants were habituated to scale-invariant sound chimeras, the other half to variable-scale ones. In each group, after habituation half of the infants were presented with change test trials (chimeras from the non-habituated category), the other half with same test trials (novel chimeras from the habituated category).

To ensure generalizability across different locations and slight variations in the experimental setup and testing room, infants were recruited at two locations: Paris and Vancouver. The procedure, the design (Habituation Type x Order) and the experimental setup were identical at both sites and results were significant irrespective of the country of origin. Infants were seated on a caregiver's lap facing a computer screen in a sound-attenuated cubicle and were tested using a habituation/dishabituation procedure [20], [21]. Under this procedure (Figure 2), participants are first habituated to sounds from one category. When their looking time drops below criterion, they are presented with new sounds that are either drawn from the other category (change trials) or from the same category (same trials). If participants detect the change in sound category, then their looking time is expected to increase in the change trials, but not in the same trials.

Caregivers listened to masking music and wore visors to avoid interference with infants' behavior. The experimenter, blind to the stimuli being presented, was seated outside the testing area, and controlled the study using the Habit X software [22]. During habituation, half of the infants were presented with scale-invariant chimeras, the other half with variable-scale chimeras (Figures 1, 2). In each 21 sec habituation trial, a different, randomly selected chimera was played, and a red-and-black checkerboard was displayed. Habituation continued until looking time across three trials decreased to criterion (65% of the first three trials). Following habituation, infants were presented with two ‘same’ trials and two ‘change’ trials. Half of the infants in each habituation group heard the same trials first, the other half the change trials first. The chimeras used for test were novel, i.e. did not appear during habituation. Infants' looking was videotaped and coded off-line. Looking times in the same and change trials were entered for data analysis.

#### Results

**Figure 3:**
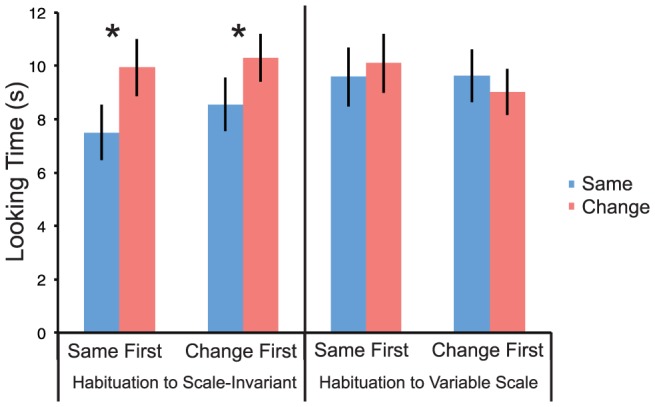
Infants' looking times to ‘same’ and ‘change’ trials. An ANOVA with Habituation Type (scale-invariant/variable-scale) and Order (same first/change first) as between-subject and Stimulus Type (same/change) as within-subject factors yielded a significant main effect of Stimulus (F(1,60) = 6.735, p = .012) and a significant Habituation Type x Stimulus Type interaction (F(1,60) = 7.419, p = .008). An ANOVA with Location (Vancouver/Paris) as an additional between-subject factor yielded similar results. To check for preference, we also conducted ANOVAs on the number of trials needed for habituation as well as on average looking times during habituation, with Habituation Type (scale-invariant/variable-scale), Order (switch first/same first) and Location (Vancouver/Paris) as between-subject factors, and found no significant effects.

An initial set of control analyses showed that the number of trials infants needed to habituate did not differ significantly between the scale-invariant (number of trials: 10.56, looking time: 11.72 sec) and variable-scale (number of trials: 11.71, looking time: 12.84 sec) habituation conditions. The overall average looking times during the habituation trials also did not exhibit a difference between the two habituation conditions (number of trials: t(31) = 1.36, ns.; looking time: t(31) = 1.49, ns.), indicating that infants did not show a preference for one category of sounds over the other, and that they had equivalent time in each condition to form a category. Importantly, however, there were differences in discrimination. Average looking times for same and change trials are shown in Figure 3. We ran an analysis of variance with Stimulus Type (same/change) as a within-subject factor and Habituation Type (scale-invariant/variable-scale) and Trial Order (same first/change first) as between-subject factors. As the main effect of the factor Testing Location was not significant, nor did the factor enter into a significant interaction, in the first analysis, we collapsed over it. There was a significant main effect of Stimulus Type (F(1,60) = 6.74, p = 0.012, partial η^2^ = 0.10), as change trials had longer looking times overall than same trials. Importantly, this was qualified by a Habituation Type X Stimulus Type interaction (F(1,60) = 7.419, p = .008, partial η^2^ = 0.11). Scheffé post hoc tests revealed that infants habituated to scale-invariant sounds (n = 32) looked significantly longer to change than to same trials (p = 0.0003), whereas infants habituated to variable-scale sounds (n = 32) showed no difference (p = 0.92, ns.). Of the 32 infants habituated to scale-invariant sounds, 25 showed longer looking times to the change than to the same trials (two-tailed binomial test: p = 0.002), whereas out of the 32 infants habituated to variable-scale sounds, 16 showed longer looking times to the change trials (two-tailed binomial test p = 1.0).

We also performed a Stimulus Type X Habituation Type ANOVA separately on each group of infants tested in the two locations to confirm the previous results and to assess the obtained effects on sample sizes more comparable to that of Experiment 1. For infants tested in Paris, the Stimulus Type X Habituation type interaction was marginally significant (F(1,30) = 3.53, p = 0.06), as infants habituated to scale-invariant sounds looked significantly longer to switch than to same trials (Scheffé post hoc: p = 0.02), but infants habituated to variable-scale sounds did not differ in their looks to the two trial types (Scheffé post hoc: p = 0.81). For infants tested in Vancouver, the main effect of Stimulus Type was significant (F(1,30) = 5.04, p = 0.032), qualified by a marginally significant Stimulus Type X Habituation Type interaction (F(1,30) = 3.97, p = 0.055), which was again due to the fact that infants habituated to scale-invariant sounds looked longer to switch than to same trials (Scheffé post hoc: p = 0.005), while infants habituated to variable-scale sounds did not (Scheffé post hoc: p = 0.86).

It is noteworthy that the infants were able to perceive and learn the category only when the scale-invariant sounds were presented in the habituation phase. This pattern of results is consistent with previous research with young infants showing that only well-formed stimuli enable perceptual anchoring and subsequent discrimination of a change [23]. This result demonstrates that it is the scale-invariance of the sounds that enables the subjects to group them in a single category. For variable-scale sounds, anchoring in a natural auditory category was not possible, thus no discrimination ensued.

#### Discussion

The above results show that 5-month-old infants can discriminate scale-invariant sounds from otherwise similar variable-scale ones. Importantly, successful discrimination was observed when infants were habituated to the natural, scale-invariant sounds, supporting the hypothesis that infants perceive scale-invariance as a natural cue for sound categorization.

Further, no difference was observed between the scale-invariant and variable-scale categories in the number and average looking time during habituation trials, implying that the results are not simply due to the more familiar nature of scale-invariant sounds, paralleling the findings of Experiment 1.

## Conclusions

Our results demonstrate that infants aged 5 months are able to learn a category of scale-invariant sounds and can discriminate them from variable-scale sounds, but cannot learn a category of variable-scale sounds. These findings suggest that the capacity to successfully recognize and categorize signals in natural auditory scenes may be rooted in infants' ability to group scale-invariant stimuli into a distinct category. That such a complex statistical feature can define a category early in infancy implies that the basis of efficient auditory coding [9] may already be found in the developing brain.

These findings have direct implications for the importance of natural stimuli, even early in life. Exposure to natural stimuli can facilitate learning and memory in adults [24]. At the behavioral level, this effect in adults has been attributed to differential allocation of attentional resources to natural stimuli [25]. Our findings are also consistent with the hypothesis that the origins of efficient learning [25] might lie in the facilitated and more automatic perception and categorization of natural as opposed to unnatural stimuli due to efficient neural coding. It will be of interest in future work to investigate this hypothesis directly, but testing whether processing advantages for other types of stimuli accrue to young infants, as they do to adults, if there is an initial exposure to natural stimuli.

Importantly, the natural world comprises not only water sounds and other sounds of nature, but also communicative sounds including human speech. It has been shown that adults are able to pull out regularities in speech even when presented at different speed/time compressions [26], suggesting that speech may have the same property of scale-invariance as do natural environmental sounds. As such, part of the privileged neural and behavioral processing of speech vs. non-speech [27], [28], [29], [30], [31] and rapid learning about the characteristics of the native language [29] may rest on the match between scale invariance in the stimuli and efficient coding. Indeed, recent computational work (REF: Lewicki 2006) suggests that certain aspects of speech might show scale-invariance. More research will be needed to establish how these properties of speech are perceived. Our novel approach, exploring the perception of the statistics of sounds created by a generative model, has the potential to place the development of auditory perception into a more general perspective. It raises the possibility that self-similarity might be a characteristic property of sounds that have biological significance, providing a unified approach to investigating how infants perceive a wide range of auditory signals from mechanical through environmental to biological and communicative sounds.
